# A focused very high energy electron beam for fractionated stereotactic radiotherapy

**DOI:** 10.1038/s41598-021-85451-8

**Published:** 2021-03-12

**Authors:** Kristoffer Svendsen, Diego Guénot, Jonas Björklund Svensson, Kristoffer Petersson, Anders Persson, Olle Lundh

**Affiliations:** 1 Department of Physics, Lund University, P.O. Box 118, 22100 Lund, Sweden; 2 Deutsches Elektronen-Synchrotron DESY, NotkestraSSe 85, 22607 Hamburg, Germany; 3 Department of Oncology, The Oxford Institute for Radiation Oncology, University of Oxford, Oxford, UK; 4 Radiation Physics, Department of Haematology, Oncology and Radiation Physics, Skåne University Hospital, Lund, Sweden

**Keywords:** Biomedical engineering, Oncology, Applied physics, Lasers, LEDs and light sources, Laser-produced plasmas

## Abstract

An electron beam of very high energy (50–250 MeV) can potentially produce a more favourable radiotherapy dose distribution compared to a state-of-the-art photon based radiotherapy technique. To produce an electron beam of sufficiently high energy to allow for a long penetration depth (several cm), very large accelerating structures are needed when using conventional radio-frequency technology, which may not be possible due to economical or spatial constraints. In this paper, we show transport and focusing of laser wakefield accelerated electron beams with a maximum energy of 160 MeV using electromagnetic quadrupole magnets in a point-to-point imaging configuration, yielding a spatial uncertainty of less than 0.1 mm, a total charge variation below $1 \%$ and a focal spot of $2.3 \times 2.6\;{\text {mm}}^2$. The electron beam was focused to control the depth dose distribution and to improve the dose conformality inside a phantom of cast acrylic slabs and radiochromic film. The phantom was irradiated from 36 different angles to obtain a dose distribution mimicking a stereotactic radiotherapy treatment, with a peak fractional dose of 2.72 Gy and a total maximum dose of 65 Gy. This was achieved with realistic constraints, including 23 cm of propagation through air before any dose deposition in the phantom.

## Introduction

Over the past decades, electron beams have mainly been used for treatment of superficial lesions due to the limited range of available electron energies, approximately 4–25 MeV^1,2^. However, studies have shown a potential for very high energy electrons (VHEE)^3^,^4^, ranging in energies from 50 to 250 MeV, in the treatment of lung cancers^5,6^, prostate cancers^6–8^, pediatric brain tumors and head and neck cancers^6^. Compared to photons, VHEE have some advantages for more deeply seated tumors. VHEE exhibits a more reliable dose deposition when there are inhomogeneities in the beam path and a sharper cut off in the dose depth profile, sparing healthy tissue more effectively^6^. Another more practical advantage is the possibility for beam steering that comes with VHEE. Due to the relatively low scattering and high penetration this makes VHEE suitable for a pencil beam electromagnetic scanning technique to irradiate the tumor. This does not require patient-specific apertures, saving time and reducing cost, and can have greater precision compared to multileaf collimators^9^. The possibility for other irradiation schemes have recently been demonstrated, such as multi-field and intensity modulation^10^. Furthermore, the low scattering may also allow for a higher dose conformality compared to photons^11^.

Achieving a VHEE beam with a traditional radio frequency-based linear accelerator requires large and costly structures as the accelerating electric fields are limited due to electrical breakdown ($<100$ MV/m). This has led to an interest in using laser-plasma based accelerators with stronger accelerating fields on the order of 100 GV/m, reducing the accelerating distance with several orders of magnitude for a given particle energy, which could result in a more compact and cost efficient solution.

Laser-plasma based accelerators can, with some modifications, accelerate different particle species, such as electrons^12^, positrons^13^, protons^14^ and ions^15^. At the current stage, only electrons are relevant for radiotherapy, as the acceleration of other particle species are limited in beam quality, having too low energy to be clinically usable because of their limited tissue penetration. One of the laser-plasma based accelerator types is the laser wakefield accelerator^12,16–19^ (LWFA). At the Lund High-Power Laser Facility, LWFA is used to accelerate electrons and is accomplished by focusing a high-power laser pulse onto a gas target. Having a high intensity at the focus, on the order of $10^{18}\;{\text {W/cm}}^2$, the leading edge of the pulse creates a plasma, a mixture of ions and free electrons, that the main part of the pulse interacts with. Due to the ponderomotive force, electrons are pushed out from high intensity regions which creates a region void of electrons inside the plasma, trailing the laser pulse like a wake. This is referred to as “the blowout regime”, in which a very strong electrostatic potential gradient is formed. Some electrons may be trapped within the wake and be accelerated by the electrostatic field over a few mm to reach hundreds of MeV.

Electron beams of different characteristics may be produced depending on the injection mechanism, i.e. the process of trapping electrons inside the plasma wake^20–25^. Common to all injection mechanisms is that the electron beam charge and energy depends on the laser pulse characteristics and, in general, a higher laser power can produce electron beams of higher charge while a higher laser energy usually results in higher electron energy. In this experiment, ionization injection^26^ was used which gives a large amount of charge (several tens to hundreds of pC), a very broad energy spectrum and an ultra short pulse duration of a few fs. Other injection schemes, such as shockfront injection, gives only a few tens of pC in charge for a 50 TW laser but a more narrow-band beam ($\Delta E \approx 5$ MeV)^23^. Ionization injection is easy to implement and one of the more robust and reproducible injection schemes which made it suitable for this experiment.

In this work, we present our advancements towards making a LWFA source suitable for fractionated stereotactic VHEE radiotherapy, i.e. the total dose is delivered in fractions where each fraction is delivered by irradiating the tumor over several different angles. Typically only one fraction per day is delivered as it allows healthy tissue to self-repair. Specifically, we report a large decrease in charge fluctuation and spatial uncertainty by using electromagnetic quadrupoles (EMQs) in a point-to-point imaging configuration. By focusing the electron beam inside a phantom using the EMQs, a more deeply penetrating beam is achieved which is used to create a 3D dose distribution similar to a typical stereotactic radiotherapy treatment.

## Method

This experiment was conducted at the Lund High-Power Laser Facility which is a titanium-doped sapphire system, having an energy on target of 600 mJ, pulse duration of 37 fs, and focused to a focal spot size of 12 $\upmu$m full width half maximum (FWHM) using an off-axis parabolic mirror with an effective focal length of 775 mm, where it reaches a peak intensity of $10^{18}\;{\text {W/cm}}^2$.

**Figure 1:**
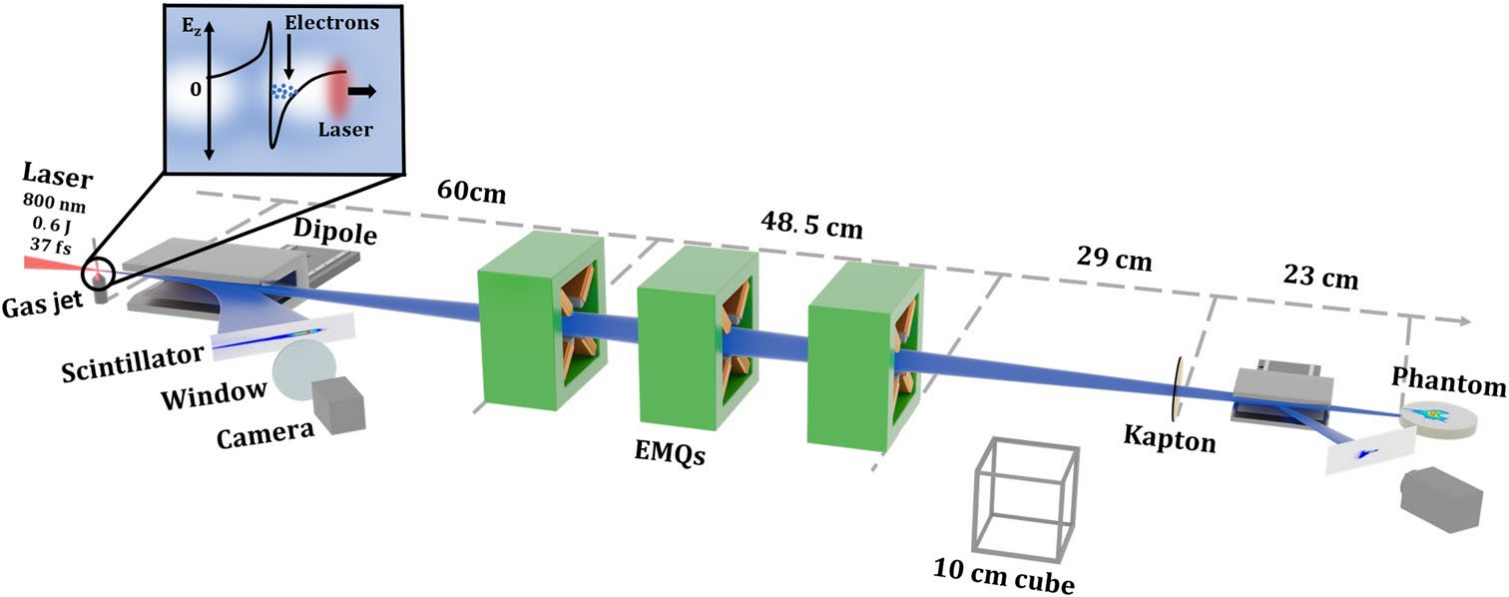
This figure shows the relevant part of the experimental setup. The laser beam enters from the left and is focused a few mm above the supersonic gas jet. The electrons are accelerated inside the plasma wake, illustrated in the inset, and propagate towards the right in the figure. A large dipole magnet on a translation stage can be positioned in the beam path to disperse the electron beam onto a scintillating screen. Following the dipole, three EMQs are positioned, set to focus 90 MeV electrons at 23 cm after the Kapton window. A smaller dipole is positioned in air to allow for a post-focused electron beam spectrum analysis along with a scintillating screen, the small dipole may also be removed to measure the electron beam profile. Finally the phantom is positioned at the electron beam focus.

The experimental setup is shown in Fig. 1. Using a supersonic gas jet with a 1.5 mm orifice, a gas mixture of helium containing $1\%$ nitrogen is ejected at a backing pressure of 4 bar which corresponds to a peak electron density of approximately $4\times 10^{18}\;{\text {cm}}^{-3}$ which, in our case, allowed for stable and reproducible ionization injected electron beams. For characterizing the electron energy spectrum before the EMQs, there is a translatable 200 mm long dipole magnet with a peak magnetic field of 0.8 T, which is used to disperse the electron beams onto a scintillating screen. The screen is imaged using a 16-bit scientific CMOS camera (Andor Zyla 4.2 Plus, Oxford Instruments, Oxfordshire, UK).

An extending vacuum pipe passes through the 3 EMQs, keeping the electron beam in vacuum until the pipe is terminated with a Kapton window, 29 cm after the last EMQ. Another scintillating screen is used to image the electron beam focus, along with a translatable 0.68 T dipole magnet to characterize the transported and focused electron beam energy spectrum. Both dipoles along with the scintillating screen were removed for radiochromic film measurements.

Gafchromic EBT3 films (Ashland Inc., Wilmington, United States) were cut into 8 cm diameter circles and 5 cm strips using scissors with care to minimize splitting of the film-edges as the films were irradiated from the side. Seven circular films were each positioned between a pair of 2 mm thick cast acrylic slabs (Perspex) in a stacked cylindrical phantom, which was then clamped to remove any air gaps which could lead to an over-response^27^. The water equivalence of cast acrylic is 1.045, i.e. 1 mm of acrylic is dosimetrically equivalent to 1.045 mm of water for an electron beam^28^.

The films were scanned 24 h after irradiation using an Epson Perfection V800 Photo scanner (Epson, Suwa, Japan) in transmission mode with no color correction, 1200 dpi and 48 bit color. The Epson V800 scanner has previously been compared to the more commonly used Epson 10000XL^29^ and shown to have 0.3% lower dose uncertainty but a stronger lateral response function, 0.2% over 8 cm and 2.7% less sensitivity on the relevant red channel^30^. The films were analyzed using the polynomial netOD method^31^ taking the red channel, calculating the dose as ${\mathcal {P}}({\text {log}}_{10}(A_{pre}/A_{post}))$ where ${\mathcal {P}}$ is the polynomial fit to the dose calibration curve and $A_{pre}$, $A_{post}$ are the red channel values of the EBT3 film pre- and post-irradiation. The calibration films ($5 \times 5$ cm) were irradiated by a clinical 10 MeV electron beam, delivered by an ELEKTA Precise linear accelerator (Elekta AB, Stockholm, Sweden), calibrated with an ion chamber according to the IAEA standard^32^. During the irradiation, the actual output was $-0.1\%$ compared to the expected output.

As previously shown, the Epson Perfection V800 Photo scanner has a more non-uniform response (i.e. uneven illumination across the scanner) due to its smaller size compared to the Epson 10000XL^30^. This was characterized and corrected by scanning large unirradiated EBT3 films, fitting a 2D cubic function which was used to correct all the scanned films.

## Results

By using EMQs, the VHEE electron beam pointing (standard deviation of the electron beam offset to the laser axis, measured 169 cm from the source) improved by more than an order of magnitude (from 1.95 to 0.09 mm), and the electron beam spatial profile a factor 3 horizontally and 2 vertically (from $7.8 \times 5.1$ to $2.3 \times 2.6$ mm, respectively). The standard deviation in photon counts from the scintillating screen, which is proportional to the total amount of charge (found by integrating the electron beam energy spectrum only considering energies above 30 MeV in both the pre- and post-focused electron beam energy spectra), was reduced by a factor 5 (from 5.16 to $0.94\%$).

**Figure 2:**
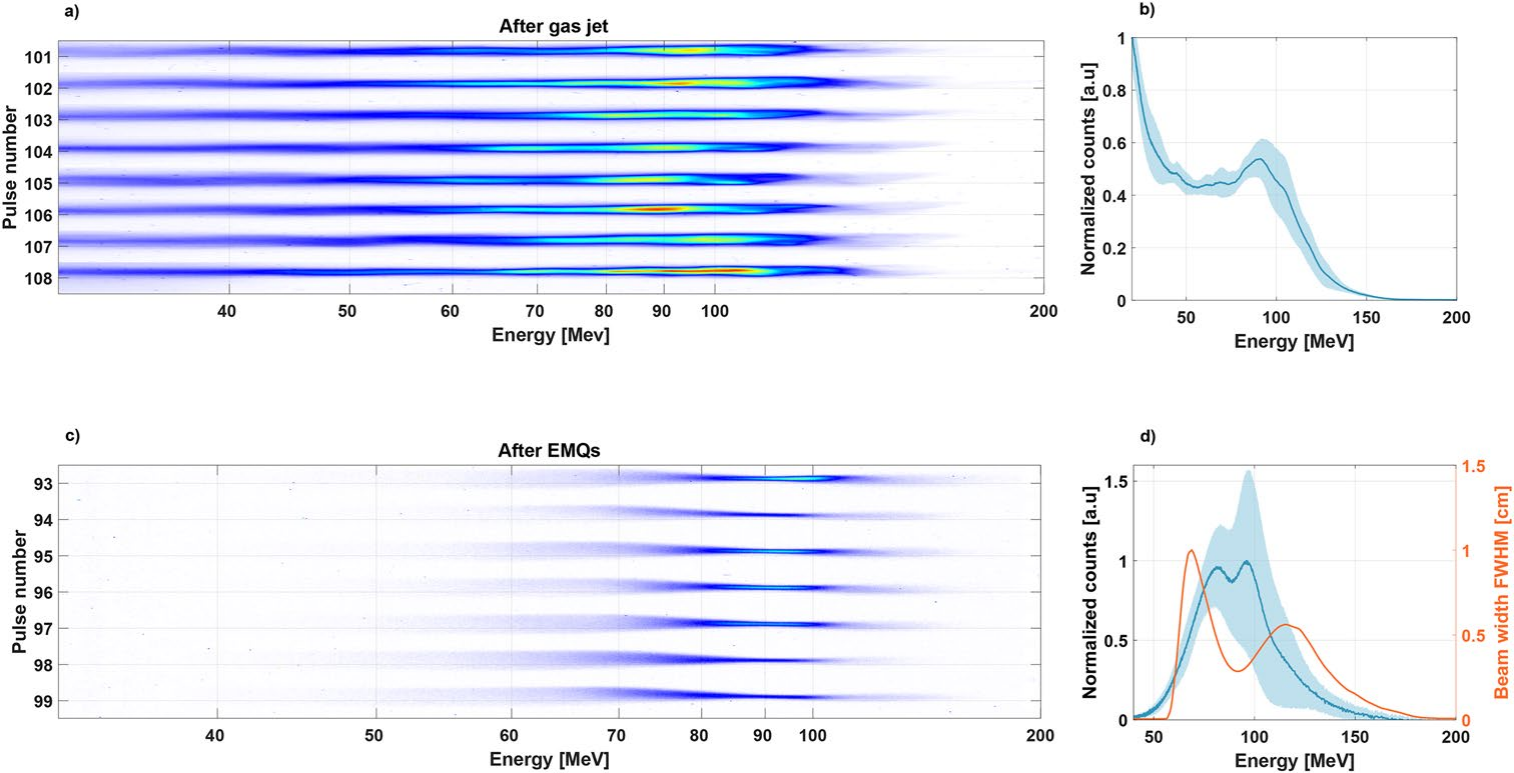
This figure shows a few of the characteristics of the electron beam. (**a**) shows 8 electron spectrometer scintillating screen images of the electron beam before focusing, with an average charge of 94 pC, (**b**) shows the average and standard deviation of the electron beam energy spectra. (**c**) Shows 7 electron spectrometer scintillating screen images of the electron beam after focusing and (**d**) shows the corresponding average electron beam energy spectra, along with the vertical FWHM of the electron beam size for pulse 97, shown in orange.

The ionization injection results in a very broad electron energy spectrum, see Fig. 2a,b. For our beam, that resulted in a maximum energy of 160 MeV with the peak in charge at the higher end of the spectrum located at 90 MeV, which was the energy the EMQs were set to focus which can be seen (in orange) in Fig. 2d. The energy spectrum from an ionization injected beam is fairly stable in charge with a low standard deviation (SD) and largely populated by electrons of 40 MeV and below. This part was however lost in the EMQs during the electron beam transport, and the resulting energy spectrum is Gaussian-like with a 40 MeV cut-off and a focus at 95 MeV, as shown in Fig. 2c,d. This energy is close to the 100 MeV that has previously been shown to be superior to state-of-the-art volumetric modulated arc therapy treatment (VMAT) plans^6^ and the pre-focused electron beam spectra peaks slightly at this energy, allowing for a focused electron beam with higher charge. The SD of the averaged focused electron beam spectra (average of seven pulses) was larger compared to the SD of the averaged electron beam before being focused (eight pulses), the SD is indicated by a blue area in Fig. 2b,d. Although the total charge remains more stable (SD of $0.94\%$), suggesting that only the shape of the spectrum is changing.

**Figure 3:**
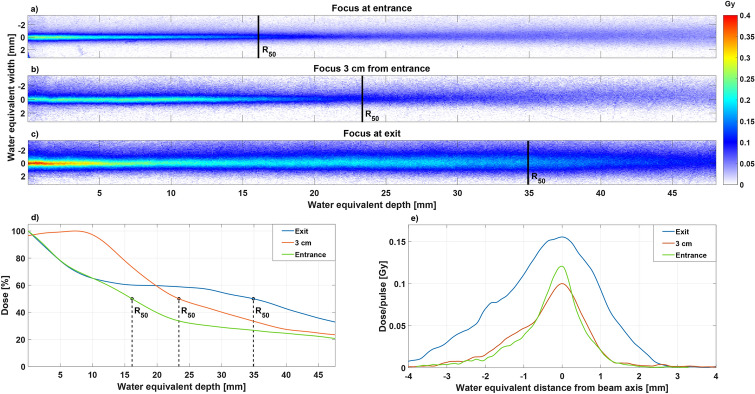
This figure shows a compilation of the features for the focused electron beam. (**a**–**c**) shows the average 2D dose profile for an ionization injected and focused electron beam pulse. The EMQs were set to focus 90 MeV at different positions; at the phantom entrance, 3 cm from the entrance and at the exit of the phantom. (**d**) Shows the percentage depth dose curves for the three focusing positions. (**e**) Shows the transverse dose profile in Gy/pulse at $R_{50}$, indicated in (**a**–**c**) as vertical lines, for the different focal positions.

By varying the focal position at the phantom (a single sheath of EBT3 clamped between a pair of 2 mm thick cast acrylic slabs, radiated from the side), the depth dose profile could be tuned to some extent. By focusing at the exit of the phantom, a higher dose may be reached in the central region, see Fig. 3a–c, which shows the average dose profiles for a phantom with varying focal position of the electron beam. For each focal length, 5 VHEE pulses were delivered. The position where $50\%$ of the max dose was delivered, $R_{50}$, was moved 14 mm deeper in the phantom by focusing at the back of the phantom, see Fig. 3d. The beam penumbra was smallest when focusing at the entrance and slightly asymmetric, 0.5 on one side of the beam axis and 0.67 mm on the other. When focusing at the exit the beam penumbra was slightly larger and more asymmetric, 1.6 mm and 0.76. When focusing at the exit, $R_{50}$ was positioned at 34.9 mm, $R_{80}$ at 4.5 and $R_{90}$ at 2.2 mm. The maximum dose was located at the phantom entrance except when focusing 3 cm from the phantom entrance, here $R_{100}$ was reached at 7.2 mm. The highest dose per pulse was reached when focusing at the phantom exit with 0.4 Gy at $R_{100}$ for a single pulse and the dose in the target area (40 mm from entrance) was 0.13 Gy per pulse. Focusing at the exit resulted in a $50\%$ higher maximum dose at $R_{50}$, with a wider lateral profile with a FWHM of 2.97 mm, compared to focusing at the entrance with a FWHM of 1.04 mm, see Fig. 3e.

**Figure 4:**
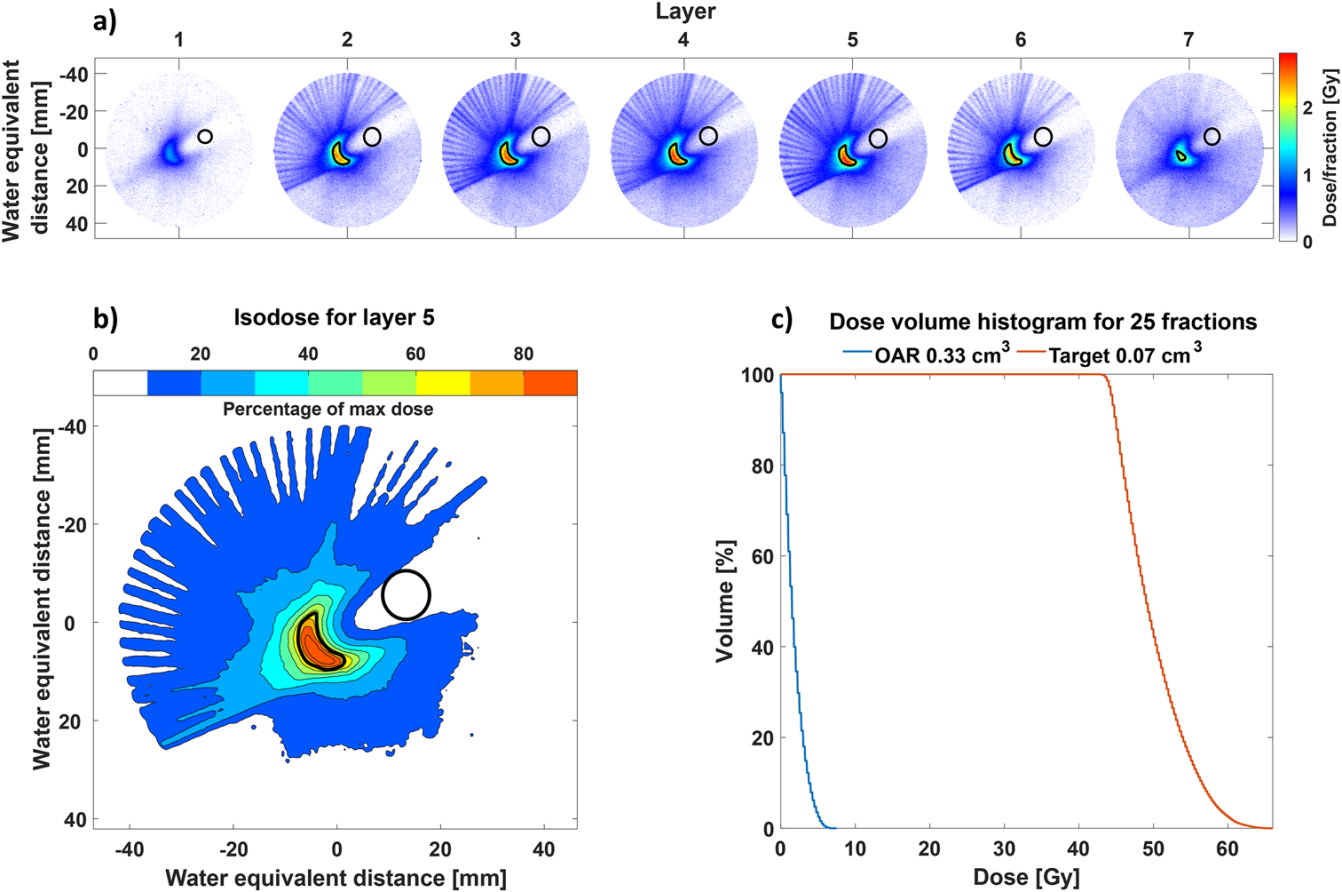
This figure shows the dosimetry data for the circular phantom. (**a**) shows the different layers of the circular phantom, 80 mm in diameter and layers 1–7 are located at height of 0, 2.1, 2.3, 2.4, 2.6, 2.7 and 4.8 mm in phantom, respectively. The target (bean-shaped) and OAR (cylinder-shaped) is outlined in black in each layer. This would correspond to a single fraction, consisting of three pulses being delivered from each of the 36 angles. The isodose for the central layer (same height as the electron beam) at 2.6 mm is shown in (**b**) along with a dose volume histogram in (**c**) for a total of 25 fractions to the target and OAR.

A cylindrical phantom consisting of Perspex with seven layers of EBT3 disks (8 cm in diameter) located at different heights (0, 2.1, 2.3, 2.4, 2.6, 2.7 and 4.8 mm) was irradiated to measure the delivered dose. To imitate a stereotactical case, a bean-shaped target volume of $0.07\;{\text {cm}}^3$ and an organ at risk (OAR) volume of $0.33\;{\text {cm}}^3$ were assumed, see Fig. 4a. The size of the phantom was chosen as a mean of relevant anatomical sites, comparable in depth and size for a head and neck case, smaller than a brain case and larger than a lung case^33^. For a typical brain metastasis stereotactical treatment^34^, the target volume is 1–$20\;{\text {cm}}^3$, 100 times larger compared the target volume in this paper, showing that our beam has sufficient precision for this treatment. Radiating a larger target volume can be achieved with the added (minor) complexity of scanning the electron beam over the target volume. The OAR was chosen to be of a size comparable to that of the optic chiasm and optical nerves for a brain case (typically located at a surface depth of less than 4 cm), the spinal cord for a head and neck case and the bronchial tree for lung cases.

Comparing the target volume in the circular phantom, see Fig. 4a, to a typical fractionated stereotactic radiotherapy treatment for meningiomas, vestibular schwannomas, pituitary adenomas or craniopharyngioma, the target volume and OAR are comparably small . Having a larger target volume would require the electron beam to be scanned over a larger area or be less focused, which is no major complication.

For a single fraction, the maximum dose was confined within the target area and reached 2.72 Gy, using 36 entrance angles with $5^{\circ }$ increments rotated around an offset of 5.2 mm from the center and three pulses from each angle. The electron beam was focused at the distal edge of the phantom to increase the dose deposition at the centrally located target. The result was a very localized dose deposition where the entrance path contained only 10–20$\%$ of the maximum dose, see Fig. 4b. The isodose curve for $70\%$ of the maximum dose was approximately confined within a circle with a diameter of 11.7 mm (not shown). For an unfocused electron beam, the circle was more than double in size, approximately 26 mm in diameter (not shown). By performing 25 fractions of this order, a total dose to the target area would reach 65 Gy, see Fig. 4c, while keeping the peak dose to the organ at risk below 8 Gy.

## Discussion

By focusing our electron beam at the end of the rectangular phantom, an increase in dose of $50\%$ at the position of $R_{50}$ was achieved while the dose for the first 10 mm remained very similar. Since the beam is polychromatic and the focal point is determined for a single electron energy (90 MeV in our experiment), all energies below 90 MeV will be focused before the focal point for 90 MeV (energies below 50 MeV will however hit the walls of the vacuum tube and be lost). This explains the higher dose seen in Fig. 3c and also why the shape of the depth dose curve for the different cases varies. The electron beam will enter the phantom converging but with lower energies converging more (at shallower depths) and higher energies less (at deeper depths). This works to offset the electron scattering inside the phantom, the lower the electron energy is, the stronger the scattering is but at the same time the focusing is stronger. Focusing at the entrance of the phantom means that all energies below 90 MeV will be diverging when entering the phantom and results in a shallower depth dose curve. As seen in Fig. 2d, the SD of the electron beam charge in the energy spectrum is increased, which is most likely caused by a slight spatial misalignment of the EMQ axis in relation the the electron beam axis and imperfect imaging conditions, resulting in increased angular fluctuations. Another, less likely, possibility would be fluctuations in the electron beam source position before the EMQs. A shift horizontally of the electron beam before the EMQs will be an offset in the horizontal direction afterwards as well, making it appear as a different energy in the spectrometer. To some extent, this would be analogous to coma in the case of an optical beam. There could also be some hysteresis present as the EMQs were not cycled.

The SD of the total amount of charge in the beams decreased from $5.16\%$ to a much more clinically acceptable level of $0.94\%$ when the beam is focused with the EMQs. As previously mentioned, this indicates that, while the shape of the energy spectrum changes (likely due to spatial misalignments) the total charge in each pulse is more stable with EMQs. This decrease is most likely due to the fact that the EMQs will only allow electrons within a certain energy band to be transmitted, the rest will be lost in the walls of the vacuum pipe. This makes for a more robust solution compared to using a dipole and an aperture to cut the electron energy spectrum, as any pointing fluctuation will translate into a charge and energy fluctuation. For a monochromatic beam, there would be less losses in the EMQs and all electrons would be focused to the same distance, resulting in an improved control in the position of $R_{50}$.

There will always be some inherent charge and spectral fluctuation, as seen in the pre-focused electron energy spectrum in Fig. 2b. However, this is random in nature so the SD will decrease as $1/\sqrt{N}$, where *N* is the total number of pulses, and thus will benefit from the larger number of pulses required for a single fraction, 108 in this case. A main concern with standard stereotactic treatment is that it takes a long time, making it difficult to keep the patient correctly positioned throughout the treatment. In this case however, the pulse repetition rate could be very high as it mainly depends on the laser system. A kHz system^35^ could deliver this fraction in 100 ms, “freezing” any potential movement of the target volume assuming it’s possible to irradiate from all angles simultaneously. Irradiating at such ultra-high dose rates would, as preclinical data has shown, have the additional benefit of a decrease in toxicity to normal tissue while maintaining the effectiveness in treating the tumor. This beneficial radiobiological effect is generally known as FLASH^36^ and has recently received a lot of attention in radiation research. To monitor the delivered dose from each pulse an integrating current transformer could be used, as the ion collection process in an ion chamber is too slow for fs electron pulses and will show an inadequate response^37^.

Further studies are necessary to investigate the long term stability^38^ and achieve better statistics of a system such as this and to further improve the VHEE beam. Another injection mechanism could be used, such as shockfront injection, which results in a more monochromatic energy spectrum. The downside of shockfront injection is that it generates less charge per TW of laser power, approximately 5.5 pC/TW of laser power above a 15 TW threshold^39^ compared to 9.2 pC/TW above 6.8 TW threshold for ionization injection^40^. Since all electrons below approximately 50 MeV are lost in the EMQs during transport anyway, this disadvantage may not be an issue as shockfront injection has a much smaller energy spread and the electron beam charge will be better preserved during beam transport and focusing. Shockfront injection produced beams do however suffer from some fluctuation in mean energy, which could be an issue during beam transport and focusing. Another possibility is self-truncated ionization injection^41^ which produces electron beams of high charge and an energy spread of only a few percent, although these beams also suffer from fluctuations in mean energy. Another possibility for lasers of moderate power, such as the one used in this study, would be to cut the desired energy from the broad ionization injection energy spectrum using a slit and focus this part. To mitigate the pointing fluctuation of the electron beam one could use a plasma lens^42,43^ to image the beam on to a slit positioned in the middle of a chicane, i.e. two dipoles to disperse the beam, followed by two more to recombine it. By imaging the beam onto the slit, the energy selection is insensitive to electron beam pointing from the source. Alternatively, solving the beam pointing at the source^44^ would make the plasma lens redundant. Once the beam is recombined at the end of the chicane it can be imaged onto a phantom using EMQs in a point-to-point imaging configuration. Having a motorized slit would allow for some additional flexibility in central energy selection and transmitted bandwidth. Although this takes an additional set of beam optics and is more complicated, it is possibly the most stable solution.

The number of patients requiring radiotherapy, in Europe alone, is expected to reach 2 million over the next 4 years^45^. As mentioned previously, VHEE plans have been shown to be similar or superior to state-of-the-art VMAT plans due to the reduced scattering, sharper penumbra and insensitive to tissue inhomogeneities, offering a significant sparing to OARs. In this comparison a VHEE beam of 100 MeV, 3 mm FWHM and emittance of 0.3$\deg$ was used^6^. A focused electron beam such as ours will further improve the dose conformality and OAR sparing due to the reduced surface and exit dose, increased dose depth and smaller electron beam size at the target.

## Conclusion

In this paper, we have shown that combining ionization injection LWFA and EMQs in a point-to-point imaging configuration can result in a clinically acceptable focused VHEE electron beam, with less than $1\%$ charge uncertainty, 0.1 mm spatial uncertainty and a spatial size of $2.3 \times 2.6\;{\text {mm}}^2$ FWHM after propagating through 23 cm of air. In addition, we have shown that the EMQs can be used to control the position of $R_{50}$ in the phantom by focusing at different longitudinal positions, moving $R_{50}$ up to 14 mm deeper.

Finally, we demonstrated the potential of our electron beam by using a cylindrical phantom to mimic a fractionated stereotactic radiotherapy treatment of a small intracranial tumor. The resulting peak fractional dose delivered to the target was 2.72 Gy which, with a total of 25 fractions, corresponds to a total maximum dose of 65 Gy to the target area while keeping the maximum dose to the OAR below 8 Gy.

We believe that this study represents a step towards clinical trials with VHEE beams generated by LWFA which can increase the availability and efficiency along with reducing the cost of radiotherapy treatment in the future.
